# Cross-Site Predictions of Readmission After Psychiatric Hospitalization With Mood or Psychotic Disorders: Retrospective Study

**DOI:** 10.2196/71630

**Published:** 2025-09-12

**Authors:** Boyu Ren, WonJin Yoon, Spencer Thomas, Guergana Savova, Timothy Miller, Mei-Hua Hall

**Affiliations:** 1 Laboratory for Psychiatric Biostatistics, McLean Hospital, Belmont, MA, United States; 2 Department of Psychiatry, Harvard Medical School, Boston, MA, United States; 3 Computational Health Informatics Program, Boston Children's Hospital, 300 Longwood Avenue, Boston, MA, 02115, United States, 1 6173556000; 4 Department of Pediatrics, Harvard Medical School, Boston, MA, United States; 5 Psychosis Neurobiology Laboratory, McLean Hospital, Belmont, MA, United States

**Keywords:** generalizability, structured data, covariate shift, inverse probability weighting, machine learning

## Abstract

**Background:**

Patients with mood or psychotic disorders experience high rates of unplanned hospital readmissions. Predicting the likelihood of readmission can guide discharge decisions and optimize patient care.

**Objective:**

The purpose of this study is to evaluate the predictive power of structured variables from electronic health records for all-cause readmission across multiple sites within the Mass General Brigham health system and to assess the transportability of prediction models between sites.

**Methods:**

This retrospective, multisite study analyzed structured variables from electronic health records separately for each site to develop in-site prediction models. The transportability of these models was evaluated by applying them across different sites. Predictive performance was measured using the *F*_1_-score, and additional adjustments were made to account for differences in predictor distributions.

**Results:**

The study found that the relevant predictors of readmission varied significantly across sites. For instance, length of stay was a strong predictor at only 3 of the 4 sites. In-site prediction models achieved an average *F*_1_-score of 0.661, whereas cross-site predictions resulted in a lower average *F*_1_-score of 0.616. Efforts to improve transportability by adjusting for differences in predictor distributions did not improve performance.

**Conclusions:**

The findings indicate that individual site-specific models are necessary to achieve reliable prediction accuracy. Furthermore, the results suggest that the current set of predictors may be insufficient for cross-site model transportability, highlighting the need for more advanced predictor variables and predictive algorithms to gain robust insights into the factors influencing early psychiatric readmissions.

## Introduction

Mood and psychotic disorders rank among the most disabling conditions worldwide [1-5]. In 2020, the United States alone spent US $186 billion on treatment for mental health disorders, constituting 6.4% of the total US health care spending [6]. This underscores the substantial economic burden of mental health conditions on the health care system. In addition, a substantial proportion of psychiatric inpatients are readmitted unplanned within 30 days after discharge [7,8]. Readmissions not only are disruptive but also cause enormous economic burden for patients and families and are a key driver of rising health care costs [9,10]. The readmission rate is considered an indicator of mental health care quality [11,12]. A 2013 US nationwide readmission study reported that psychiatric disorders account for the largest portion (24%) of short-term (<30-day) readmissions among adults between 18 and 44 years old; that the readmission rate of patients with schizophrenia is among the highest (25.7%); and that psychiatric disorders, including substance abuse, contribute to one-tenth of all discharges leading to readmission, with nearly one-fifth of discharges leading to readmission occurring in Medicare- and Medicaid-insured individuals [13]. Addressing and predicting unplanned readmission are therefore major unmet needs of psychiatric care.

Previous studies leveraging structured elements of electronic health record (EHR) data for short-term psychiatric readmission prediction have identified various risk factors associated with 30-day unplanned psychiatric readmissions, including gender, marital status, insurance status, specific diagnoses (eg, schizophrenia), substance use, and length of stay (LOS) [7,14]. More recently, natural language processing (NLP) techniques have been employed to extract insights from unstructured clinical notes for readmission risk prediction. These studies corroborated findings from structured elements while uncovering additional features such as suicidality [15], prior psychiatric admissions [16], family relationships [16,17], and symptom severity (eg, mood and psychosis) [16-18].

Despite the potential of EHR data in readmission prediction, several limitations persist. One important issue is the scarcity of cross-dataset validation. Most studies focus on model development and validation within a single hospital or a dataset drawn from a large health care system consisting of different hospitals. For the latter, it is assumed that hospital-specific differences in practices, documentation, and patient populations within the same health care system are largely negligible. However, each hospital might have a history of serving specific patient populations and psychiatric needs. True cross-dataset validation, in which a model trained on one hospital dataset is explicitly tested on another, is exceedingly rare. This limitation hampers our understanding of how well prediction models and predictive variables generalize across different hospitals and patient populations. Furthermore, direct comparisons of various prediction algorithms across independent datasets are uncommon, limiting our understanding of how models might perform in diverse settings with various patient populations or institutional patient care practices. These issues are important considerations for building robust and widely applicable readmission prediction models.

To address these issues, we extracted psychiatric inpatient EHR data from 4 different hospitals within the same health care system in the Boston area. We focused on assessing characteristics of patient populations across hospitals, performing cross-dataset validation in which a model trained on one hospital dataset was tested on another, and comparing various prediction algorithms across independent hospital datasets. This study used data that are widely available and standardized across health systems to establish a baseline for readmission prediction models. These models provide a transparent and reproducible benchmark against which future models, particularly those incorporating additional information sources such as unstructured text or claims data, can be compared.

## Methods

### Study Design and Cohort Generation

This is a retrospective study of patients with EHRs from inpatient psychiatric unit stays at 4 Boston academic medical hospitals within the Mass General Brigham (MGB) system: Massachusetts General Hospital (MGH), McLean Hospital (MCL), Brigham and Women’s Hospital (BWH), and Faulkner Hospital (FH). EHRs were sourced from the Research Patient Data Registry, a centralized regional data repository that serves all institutions within the MGB health care system. While these 4 hospitals have different patient populations and staff and operate largely independently, their records are all accessible in the Research Patient Data Registry system.

We queried the EHRs of patients, separately in each hospital, who were aged between 18 and 65 years at the time of query (April 2023) and with any diagnosis codes for mood disorders (F30-F39 in the *International Statistical Classification of Diseases, Tenth Revision* [*ICD-10*]) or psychotic disorders (F20-F29 in *ICD-10*). While *ICD* codes may have low positive predictive value for phenotyping studies [19], for this work, we applied liberal inclusion criteria to take a broader view of these disorders and increase the size of our labeled datasets. Structured data included demographics (age, sex, race/ethnicity, insurance type, and diagnoses). The dataset also included unstructured clinical narratives (progress reports and discharge summaries), the analysis of which will be described in future work.

### Data Extraction

To study all-cause readmission, we extracted the set of patients with inpatient encounters matching the above inclusion criteria and then all additional inpatient encounters for those patients across MGB sites. The i2b2 [20] query interface at MGB allowed us to specify these criteria in a graphical user interface. The query results were returned as text files representing database tables, with 1 row per encounter. To create classification instances, we automatically labeled inpatient encounters as being “psychosis or mood disorder related” if they contained an *ICD-10* billing code starting with F2 or F3. We then collected the set of additional encounters for these patients and ordered all encounters by date. The database column contained an “Inpatient/Outpatient” column, which we used to define inpatient encounters. Inpatient encounters that overlapped in dates were merged during preprocessing. These merged inpatient encounters represent the classification instances. For each classification instance, it was given a positive label (representing early readmission) if the admission date of the next inpatient encounter in the patient encounter sequence was within 30 days of the discharge date of the discharge encounter.

### Prediction Algorithms and Variables

We considered two models for the prediction of 30-day readmission: logistic regression and random forest (RF). Logistic regression is the most widely used classification model with a linear decision boundary, while RF allows for a nonlinear decision boundary and can handle complex interactions between predictors. For both models, we used age at discharge; sex; race; diagnosis of psychosis, mood disorder, anxiety, and substance use disorder (SUD); and LOS (in days) as the predictors. We included only the main effects of the predictors in the logistic regression model for simplicity. We then evaluated the within-site and cross-site prediction accuracy of these models. For each site, we split the original data into training and testing sets in a 3-to-1 ratio. To create these splits, we used the last digit of a cross-site patient identifier so that patients with encounters at more than one site were in the same split at different sites (ie, a patient would not be in the training set at MCL and the testing set at MGH).

To account for differences in sample sizes across sites, we trained each site-specific model using a subsampled training set matched to the smallest training sample size among all sites (n=1053). The site-specific models were then applied to the testing set of the same site for within-site evaluation and the testing sets of other sites for cross-site evaluation. We repeated the subsampling procedure 100 times and used the average area under the receiver operating characteristic (ROC) curve (AUC) and *F*_1_-score for readmission across replications to measure prediction performance. We calculated *F*_1_-scores using the optimal threshold that maximizes the sum of the squared sensitivity and specificity based on the ROC curve.

In Multimedia Appendix 1, we report results from an additional analysis in which 4 socioeconomic predictors, derived from names of insurance and residential zip code, were added to the models to assess whether incorporating this information could further improve predictive performance.

### Cross-Site Prediction With Covariate Shift

The in-site prediction model for site s is designed to optimize the expected prediction performance with respect to the data distribution of site s. When applying it to a different site, it might not be able to produce useful predictions if the data distribution changes. With the aim to improve the cross-site prediction performance, we used a simple inverse probability weighting (IPW) strategy to account for a particular type of shift in data distributions across sites (ie, covariate shift), which assumes that the only difference in data distributions is in the marginal distributions of the predictors, whereas the conditional distribution of the outcome given the predictors remains idennetical across sites [21]. This method aligns the distributions of the predictors at a source site and a target site with a weighting approach such that the in-site prediction model based on the weighted samples in the source site minimizes the expected prediction performance with respect to the data distribution of the target site. Specifically, when aligning the distribution of predictors X in site s with that in site s`, we merged the two datasets and fit a logistic regression model to estimate the conditional probability $Pr\left(site=s^{\prime }|X\right)$. The weight assigned to sample i in site s was then calculated as $\frac{\mathrm{Pr}\left(site=s^{\prime }|X_{i}\right)}{1-\mathrm{Pr}\left(site=s^{\prime }|X_{i}\right)}$. Intuitively, this weighting scheme increases the influence of individuals in site s whose covariate profiles resemble those more common in site s`. For example, if site s` had a larger proportion of females than site s, then female subjects in site s would be up-weighted and males down-weighted in model training. For a more formal description of this method, see Multimedia Appendix 2. In our analysis, the predictors include age at discharge, sex, race, LOS, and the binary indicators for psychosis, mood disorder, anxiety, and SUD.

#### Ethical Considerations

This study was approved by the MGB Institutional Review Board (2022P000181). This is an MGB protocol with a reliance at Boston Children’s Hospital (IRB-P00042305). The institutional review board did not require informed consent because this was a retrospective study of a large cohort using data acquired during clinical care. The data for this study does not contain identifiers. The larger dataset it was extracted from does contain identifiers. It is stored on HIPAA (Health Insurance Portability and Accountability Act)–compliant network drives inside the MGB firewall, and all analysis was done within that environment. The data is only accessible to investigators who are listed on the institutional review board approval and have completed training in human subjects research. Subjects were not compensated, as this is a retrospective study on EHR data that was collected in the course of care.

## Results

### Summary Statistics of the Dataset

Table 1 shows a detailed breakdown of psychiatric inpatient encounters (ie, classification instances), their breakdown across sites, and the statistics of the variables associated with them. The initial query and postprocessing yielded 52,237 classification instances across 4 sites: MCL (n=29,845), MGH (n=18,772), BWH (n=1406), and FH (n=1053). Based on Table 1, no pair of sites shares similar distributions across all predictors. A clustering structure is evident for certain predictors. For example, records in BWH and FH have similar racial composition, while MCL and MGH share the same racial distribution. However, the clustering structure varies across different predictors. A patient may have multiple F2 or F3 diagnoses, or both, in a single encounter; most of the encounters had a diagnosis of mood disorders (MCL had the lowest prevalence: 22,443/29,845, 75.2%). Diagnosis of psychosis, anxiety, and SUD was less common. The proportion of 30-day readmission was not too extreme in all four sites, ranging from 21.1% (222/1053) in FH to 43.3% (8129/18,772) in MGH.

**Table 1.** Distributions of predictors in consideration and the outcome (30-day readmission) in four study sites.

	BWH^a^ (n=1406)	Faulkner (n=1053)	MCL^b^ (n=29,845)	MGH^c^ (n=18,772)
LOS^d^				
Mean (SD)	8.85 (19.3)	12.9 (17.1)	12.0 (50.5)	9.39 (15.6)
Median (IQR)	5.00 (3.00-9.00)	8.00 (6.00-14.0)	8.00 (5.00-14.0)	6.00 (2.00-12.0)
Legal sex, n (%)				
Female	803 (57.1)	461 (43.8)	16,340 (54.7)	8985 (47.9)
Male	603 (42.9)	592 (56.2)	13,496 (45.2)	9786 (52.1)
Missing	0 (0.0)	0 (0.0)	9 (0.0)	1 (0.0)
Race, n (%)				
White	841 (59.8)	627 (59.5)	24,341 (81.6)	15,164 (80.8)
Black	321 (22.8)	230 (21.8)	1522 (5.1)	1567 (8.3)
Asian	11 (0.8)	11 (1.0)	690 (2.3)	310 (1.7)
Other	148 (10.5)	141 (13.4)	981 (3.3)	1166 (6.2)
Missing	85 (6.0)	44 (4.2)	2311 (7.7)	565 (3.0)
Age at discharge (years)				
Mean (SD)	41.4 (12.6)	41.4 (13.2)	34.9 (11.4)	45.7 (12.1)
Median (IQR)	42.0 (30.0-52.0)	41.0 (29.0-53.0)	34.0 (24.0-44.0)	48.0 (38.0-56.0)
Psychosis, n (%)				
Yes	294 (20.9)	404 (38.4)	6609 (22.1)	4317 (23.0)
No	1112 (79.1)	649 (61.6)	23,236 (77.9)	14,455 (77.0)
Mood, n (%)				
Yes	1255 (89.3)	823 (78.2)	22,443 (75.2)	16,830 (89.7)
No	151 (10.7)	230 (21.8)	7402 (24.8)	1942 (10.3)
Anxiety, n (%)				
Yes	300 (21.3)	213 (20.2)	10,951 (36.7)	5599 (29.8)
No	1106 (78.7)	840 (79.8)	18,894 (63.3)	13,173 (70.2)
SUD^e^, n (%)				
Yes	591 (42.0)	477 (45.3)	14,121 (47.3)	8394 (44.7)
No	815 (58.0)	576 (54.7)	15,724 (52.7)	10,378 (55.3)
Outcome				
Mean (SD)	0.329 (0.470)	0.211 (0.408)	0.283 (0.450)	0.433 (0.496)

a BWH: Brigham and Women’s Hospital.

b MCL: McLean Hospital.

c MGH: Massachusetts General Hospital.

d LOS: length of stay.

e SUD: substance use disorder.

### In-Site Predictions

The estimated regression coefficients of the logistic model for all sites are illustrated in Table 2. None of the predictors were consistently significant across all sites, and the estimated odds ratio can be qualitatively different from site to site. LOS was significantly associated with the outcome in 3 sites, but with different directionality (negative association in MGH and positive associations in BWH and FH). Sex and diagnosis of anxiety and SUD were significantly associated with the outcome in MGH and MCL, although the directionality of the association of diagnosis of anxiety was different between these two sites. Age at discharge only showed significance in BWH, and the contrast between other races and White, as well as the diagnosis of psychosis and mood disorders, only showed significance in MGH.

**Table 2.** Estimated regression coefficients of the logistic models for each of the four sites. Note: The regression coefficients are transformed into the scale of odds ratios. The reference level of race is “White.”

	MGH^a^		MCL^b^		BWH^c^		FH^d^	
Predictors	Odds ratio (95% CI)	*P* value	Odds ratio (95% CI)	*P* value	Odds ratio (95% CI)	*P* value	Odds ratio (95% CI)	*P* value
Age at discharge	1.001 (0.999-1.004)	.25	1.002 (1.000-1.005)	.26	0.987 (0.977-0.998)	*.02^e^*	0.999 (0.985-1.013)	.70
Legal sex (male)	1.400 (1.303-1.504)	*<.001*	1.104 (1.037-1.175)	*.002*	1.144 (0.863-1.517)	.40	1.012 (0.697-1.475)	.92
Race (Black)^f^	0.952 (0.836-1.083)	.45	0.930 (0.813-1.061)	.36	1.232 (0.891-1.698)	.30	1.105 (0.684-1.756)	.81
Race (Asian)^f^	0.785 (0.590-1.040)	.15	1.065 (0.882-1.282)	.40	1.509 (0.366-5.826)	.55	1.839 (0.386-6.848)	.29
Race (Other)^f^	0.765 (0.661-0.883)	*.001*	0.941 (0.795-1.111)	.54	1.316 (0.843-2.033)	.23	0.921 (0.515-1.589)	.76
Psychosis (TRUE)	0.493 (0.439-0.553)	*<.001*	0.93 (0.852-1.015)	.07	1.047 (0.650-1.657)	.82	1.394 (0.837-2.279)	.16
Mood (TRUE)	0.598 (0.514-0.696)	*<.001*	1.025 (0.944-1.114)	.58	1.026 (0.563-1.861)	.89	1.123 (0.631-1.990)	.72
Anxiety (TRUE)	0.631 (0.583-0.682)	*<.001*	1.095 (1.029-1.165)	*.01*	0.773 (0.541-1.092)	.15	1.307 (0.825-2.035)	.28
SUD^g^ (TRUE)	0.403 (0.375-0.433)	*<.001*	0.627 (0.588-0.668)	*<.001*	1.084 (0.820-1.431)	.52	1.006 (0.686-1.474)	.96
LOS^h^	0.981 (0.978-0.984)	*<.001*	1.000 (1.000-1.001)	.34	1.013 (1.005-1.023)	*.007*	1.010 (1.001-1.019)	*.04*

a MGH: Massachusetts General Hospital.

b MCL: McLean Hospital.

c BWH: Brigham and Women’s Hospital.

d FH: Faulkner Hospital.

e Italic*s* indicate *P*<.05.

f White was used as the reference for the race category.

g SUD: substance use disorder.

h LOS: length of stay.

The results of the feature importance study using RF are shown in Table 3. The feature importance, measured by the decrease in prediction accuracy when randomly permuting a variable while keeping all other variables intact, reveals a different landscape of predictive power associated with each predictor. For all four sites, LOS had the highest importance among all predictors. However, the actual magnitude of this predictor was quite small except in the case of MGH (0.175). All other predictors were much less predictive, with the highest importance achieved for SUD at 0.016. Note that the accuracy of a prediction model ranges from 0 to 1. A decrease of more than 0.1 can be considered a substantial drop in the model performance, while a decrease of less than 0.01 is likely negligible.

**Table 3.** Feature importance of all predictors, measured by the decrease in accuracy when removing the variable from the random forest across all four sites. Larger positive values indicate higher importance.

Variable	MGH^a^ (Acc^b^=0.765)	MCL^c^ (Acc=0.745)	BWH^d^ (Acc=0.656)	FH^e^ (Acc=0.814)
Age at discharge	0.0072	0.0022	0.0050	0.0030
Legal sex	0.0052	0.0009	–0.0016	0.0000
Race	0.0033	0.0002	0.0042	0.0033
Psychosis	0.0093	0.0200	0.0011	0.0042
Mood	0.0032	0.0152	0.0024	0.0020
Anxiety	0.0067	0.0033	–0.0018	0.0034
SUD^f^	0.0161	0.0130	0.0004	0.0001
LOS^g^	0.1752	0.0292	0.0080	0.0085

a MGH: Massachusetts General Hospital.

b Acc: out-of-bag estimate of the accuracy of the model with all variables included.

c MCL: McLean Hospital.

d BWH: Brigham and Women’s Hospital.

e FH: Faulkner Hospital.

f SUD: substance use disorder.

g LOS: length of stay.

**Figure 1.**
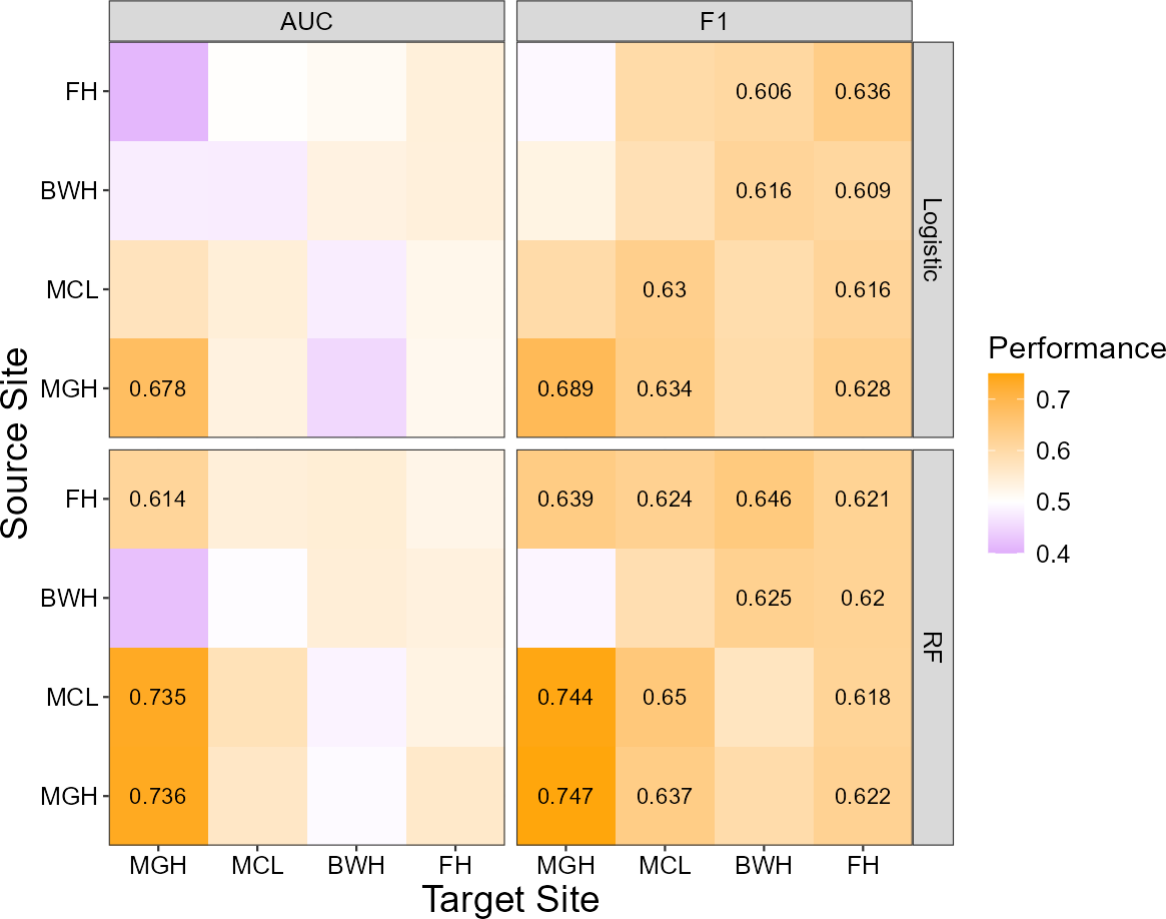
In- and cross-site prediction performance of the logistic regression models (top) and RF models (bottom). Only AUC and *F*_1_-scores greater than 0.6 are displayed as text in the figure. AUC: area under the receiver operating characteristic curve; BWH: Brigham and Women’s Hospital; FH: Faulkner Hospital; MCL: McLean Hospital; MGH: Massachusetts General Hospital; RF: random forest.

The AUCs and *F*_1_-scores of the logistic regression models applied to the testing data of the same sites are shown in the top panels of Figure 1 (diagonal elements of the heatmaps). Except for MGH, none of the sites achieved an AUC larger than 0.6, indicating a low level of discriminative power of these models. MGH also had the highest *F*_1_-score among all sites. The results for RF are shown in the bottom panels of Figure 1. Only MGH showed a discernible improvement in prediction performance when switching from the logistic regression model to RF—the AUC for MGH increased from 0.678 to 0.736, and the *F*_1_-score increased from 0.689 to 0.747. All results combined suggest that the predictors included were suitable only for the prediction of 30-day readmission for encounters at MGH.

### Cross-Site Predictions

When a prediction model trained on data from one site was applied directly to testing data from another site, the prediction performance was generally worse than in-site predictions (see Figure 1, off-diagonal elements), demonstrating a general lack of transportability of the prediction models across sites. The only exception was MGH. RF models from FH and MCL both achieved reasonable AUCs and *F*_1_-scores when tested on MGH data. The RF model trained with MCL data even produced an AUC nearly identical to the in-site predictions for MGH. This indicates that the MGH records are inherently easier to predict. The results were largely unchanged after adding the 4 socioeconomic status (SES) predictors to the model (see Multimedia Appendix 1 for details). The only notable improvement was observed in the logistic regression model for MGH, in which the within-site prediction improved the AUC from 0.678 to 0.706 and the *F*_1_-score from 0.689 to 0.728.

**Figure 2.**
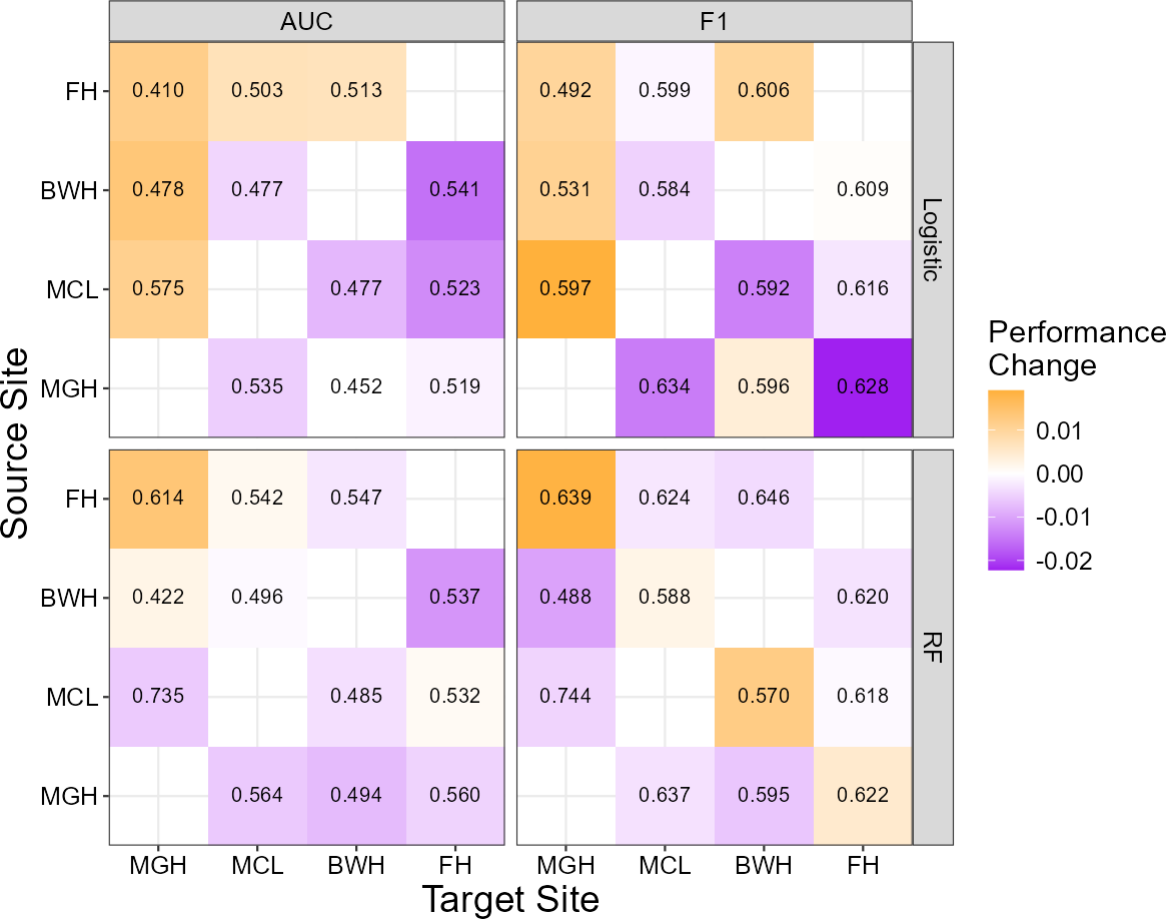
Cross-site prediction performance of the logistic regression models (top) and RF (bottom) after adjusting for covariate shift. Text values represent model performance with covariate shift adjustment, while tile colors indicate the magnitude and direction of the adjustment’s effect: positive changes in the prediction metric are shown in orange and negative changes in purple. AUC: area under the receiver operating characteristic curve; BWH: Brigham and Women’s Hospital; FH: Faulkner Hospital; MCL: McLean Hospital; MGH: Massachusetts General Hospital; RF: random forest.

One potential reason for this observed lack of transportability was covariate shift, that is, the distribution of the predictors differed from site to site, as confirmed in Table 1. The performance of cross-site predictions that adjusted for covariate shift, based on the IPW approach introduced in the Methods section, is illustrated in Figure 2. The results clearly show that adjustment for covariate shift has relatively low benefit to the transportability of the prediction models, as nearly none of the AUCs or *F*_1_-scores improved substantially after weighting. Adding the 4 SES predictors had no discernible impact on the performance of the IPW approach (see Figure S2 in Multimedia Appendix 1).

## Discussion

This study focused on assessing characteristics of patient populations across four hospitals, performing cross-dataset validation, and comparing two prediction algorithms, logistic regression and RF, across independent hospital datasets. Our results showed that the distribution of the predictors differed across all sites and that the prediction of readmission risk using structured EHR data is a very challenging task. Our results further highlight substantial variability across sites in both predictive performance and the feature importance of input variables, even though all four hospitals belong to the same health system, share databases, and are located in the same geographic area. Fitted models from both algorithms showed that LOS is an important feature of readmission risk. In the logistic regression model, LOS is significantly associated with the outcome in 3 sites (Table 2), and in the RF model, it has the highest importance among all predictors (Table 3). Longer LOS predicts greater readmission risk in BWH and FH. The unexpected negative association observed at MGH may be explained by the presence of heterogeneous subpopulations within its cohort, potentially because of its large sample size. For instance, a subset of patients with milder symptoms may have stabilized quickly and been discharged after a short stay, yet still experienced early relapse due to insufficient stabilization or support, thereby increasing their likelihood of readmission. Conversely, patients with more severe conditions may have benefited from a longer hospitalization that allowed for more comprehensive treatment and discharge planning, thereby reducing their immediate readmission risk. This could result in a nonlinear, possibly U-shaped relationship between LOS and readmission at MGH, with the observed LOS distribution primarily falling within the decreasing segment of the curve. This variability in the LOS associations, together with the lack of significant associations for diagnosis-related predictors at BWH and FH, likely due to limited sample size rather than a true absence of effect, highlights the challenges in generalizing site-specific models.

We also showed that the most straightforward explanation for the lack of cross-site transportability, *covariate shift*, did not account for much of the performance difference. Although there were significant differences in input variable distributions between sites, the negligible or even negative effect of our inverse probability weighting strategy suggests that more complex modes of heterogeneity across sites are present. For example, patients’ comorbidities not related to mental health may interact with mental health conditions in complex ways, resulting in different conditional distributions in each site. As a result, modeling covariate shift using a simple reweighting strategy in this study was not able to capture a complex heterogeneity structure. The sites varied in multiple ways. The sample sizes of MCL and MGH were significantly larger than BWH and FH. The smaller sample sizes at BWH and FH may have limited the ability to detect subtle site-specific effects and potentially masked meaningful associations that might otherwise have been observed in larger datasets. In addition, MCL is a dedicated psychiatric facility, whereas for the other 3 sites, psychiatry is just one of many specialties. It is currently unknown to what extent the diagnostic input codes from EHRs relate to the readmission risk in a different way at different sites because of the heterogeneity of the patient profiles linked to different sites—for example, perhaps a site like MCL is seeing patients with more complex medical and psychiatric comorbidities. Additionally, variability in *ICD* coding practices across hospitals may further alter the observed effects of specific predictors, introducing another layer of site-specific variation that complicates cross-site generalizability.

Although these site-level characteristics are clearly relevant to cross-site generalizability, they cannot be directly incorporated into site-specific models, as all individuals within a site share the same values for these variables. However, such institutional differences may give rise to variation in predictive but unmeasured predictors for readmission risk, as well as modify the effects of observed predictors. For example, psychiatric specialty hospitals like MCL are more likely to treat patients with greater disease severity or complex psychiatric and medical comorbidities. These latent features, though not captured in structured EHR data, may largely drive the influence of site-level context on predictive performance. Extracting such features through NLP applied to unstructured clinical text represents an important next step. This approach could enable more accurate adjustment for underlying patient complexity, reducing the need to account explicitly for site-level characteristics and ultimately improving both the predictive performance and transportability of site-specific models.

This study intentionally focused on structured EHR variables, such as demographics, diagnostic codes, and visit data, to provide a baseline for readmission prediction using readily accessible and standardized data sources. Although we acknowledge that these features showed limited predictive power, they are often the most scalable and widely available across health care systems. Additional features relevant to readmission risk, such as outpatient follow-up, medication adherence, and SES, can in principle be approximated using structured EHR data; however, they are often inconsistently or incompletely recorded, offering minimal added predictive power (see Multimedia Appendix 1 for more details). These baseline models enable a clear assessment of the added predictive value of complex features derived through more advanced feature engineering techniques. We also recognize that unstructured clinical notes capture richer details. Variables derived from unstructured clinical text are likely to provide greater predictive value because of their increased granularity and contextual richness. For example, for the hypothesis above concerning severity and comorbidities, the proposed NLP-based approach could help classify psychiatric patients based on the presence of comorbidities not related to mental health and support more accurate modeling of illness severity. Ongoing work is pursuing this direction [22].

Finally, we noted that the lack of transportability in predicting readmission seemed to be less prominent between MCL and MGH—when the in-site RF model of either hospital was applied to the other, the resulting performance was comparable to that of the in-site predictions. One possible explanation for this anomalous result, as indicated by the feature importance in Table 3, is that the RF models for both hospitals share the same dominating predictor, LOS, which accounts for a nonignorable proportion of the outcome variability. It is likely that there are unobserved yet highly predictive variables that are unique to each site, and by including them in the models, the in-site predictions could be improved such that the anomaly observed here for MCL and MGH is no longer present. This finding again highlights the necessity of moving beyond the current set of structured variables for predictions of readmission.

## Supplementary material

10.2196/71630 Multimedia Appendix 1 Results of the supplementary analysis incorporating four additional socioeconomic status variables (insurance type, median household income, poverty rate, and educational attainment) into the models.

10.2196/71630 Multimedia Appendix 2 Details of the inverse probability weighting approach used to adjust for covariate shift.

## Abbreviations

AUC: area under the receiver operating characteristic curve
BWH: Brigham and Women’s Hospital
EHR: electronic health record
FH: Faulkner Hospital
HIPAA: Health Insurance Portability and Accountability Act
LOS: length of stay
MCL: McLean Hospital
MGB: Mass General Brigham
MGH: Massachusetts General Hospital
NLP: natural language processing
RF: random forest
ROC: receiver operating characteristic
SES: socioeconomic status
SUD: substance use disorder
