# Supplementary material for: Cross-Site Predictions of Readmission After Psychiatric Hospitalization With Mood or Psychotic Disorders: Retrospective Study
Source: JMIR Ment Health. 2025 Sep 12;12:e71630. doi: 10.2196/71630 (PMC12431164; doi:10.2196/71630)
Supplement: Multimedia Appendix 1 [file mental-v12-e71630-s001.docx]

#### Incorporation of Socioeconomic Status (SES) Variables

As a supplementary analysis, we incorporated four additional variables as proxies for SES. Two types of SES-related information were derived from patients’ insurance information and residential ZIP codes. First, insurance type was categorized as commercial or non-commercial using a rule-based algorithm that flagged insurance plan names containing keywords such as “Medicare,” “Medicaid,” “MassHealth,” “Free,” “Unicare,” “Commonwealth,” as well as references to state or city programs, as non-commercial. This classification served as a coarse proxy for individual-level income.

Second, we used patients’ ZIP codes to link to neighborhood-level socioeconomic indicators from the U.S. Census. From these data, we extracted median household income, poverty rate, and the percentage of residents with a bachelor’s degree or higher, three commonly used measures of community-level SES. These variables were used to assess whether the inclusion of SES information improved the predictive performance of readmission models.

Incorporating the four SES-related variables resulted in minimal changes to overall model performance. A modest improvement in predictive performance was observed only for the logistic regression model trained on the MGH site. For all other models and sites, the inclusion of SES proxies did not yield meaningful gains in accuracy or discrimination (Figure S1). Importantly, the addition of these variables did not substantially affect model transportability across sites (Figure S2), suggesting that the SES-related information, as operationalized in this study, did not meaningfully alter the generalizability of the models.


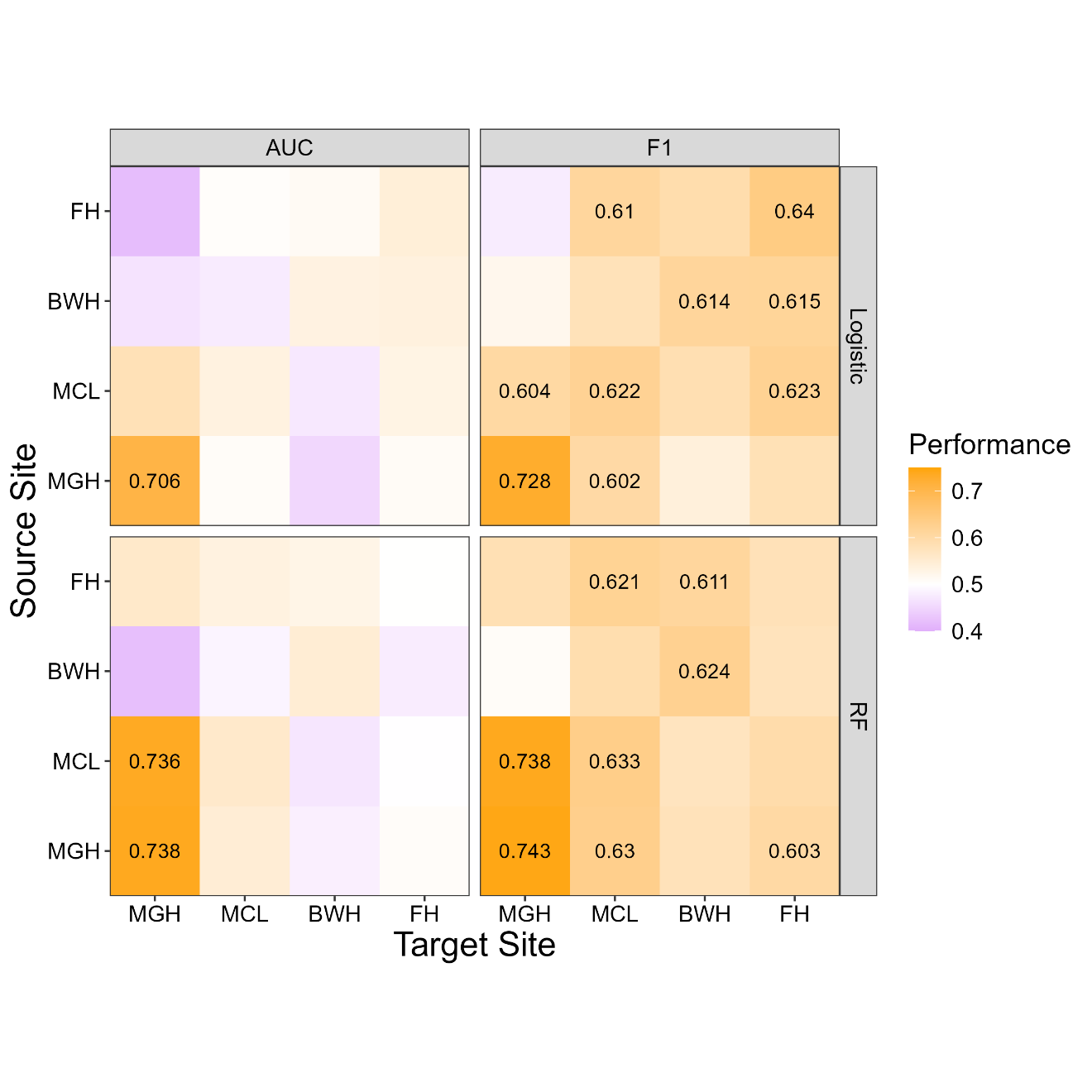


Figure S1: In- and cross-site prediction performance of the logistic regression models (top) and RF (bottom) with four additional SES variables included. Only AUC and F1 scores greater than 0.6 are also displayed as text in the figure. BWH=Brigham and Women’s Hospital, MCL=McLean, MGH=Mass General Hospital, FH=Faulkner Hospital.


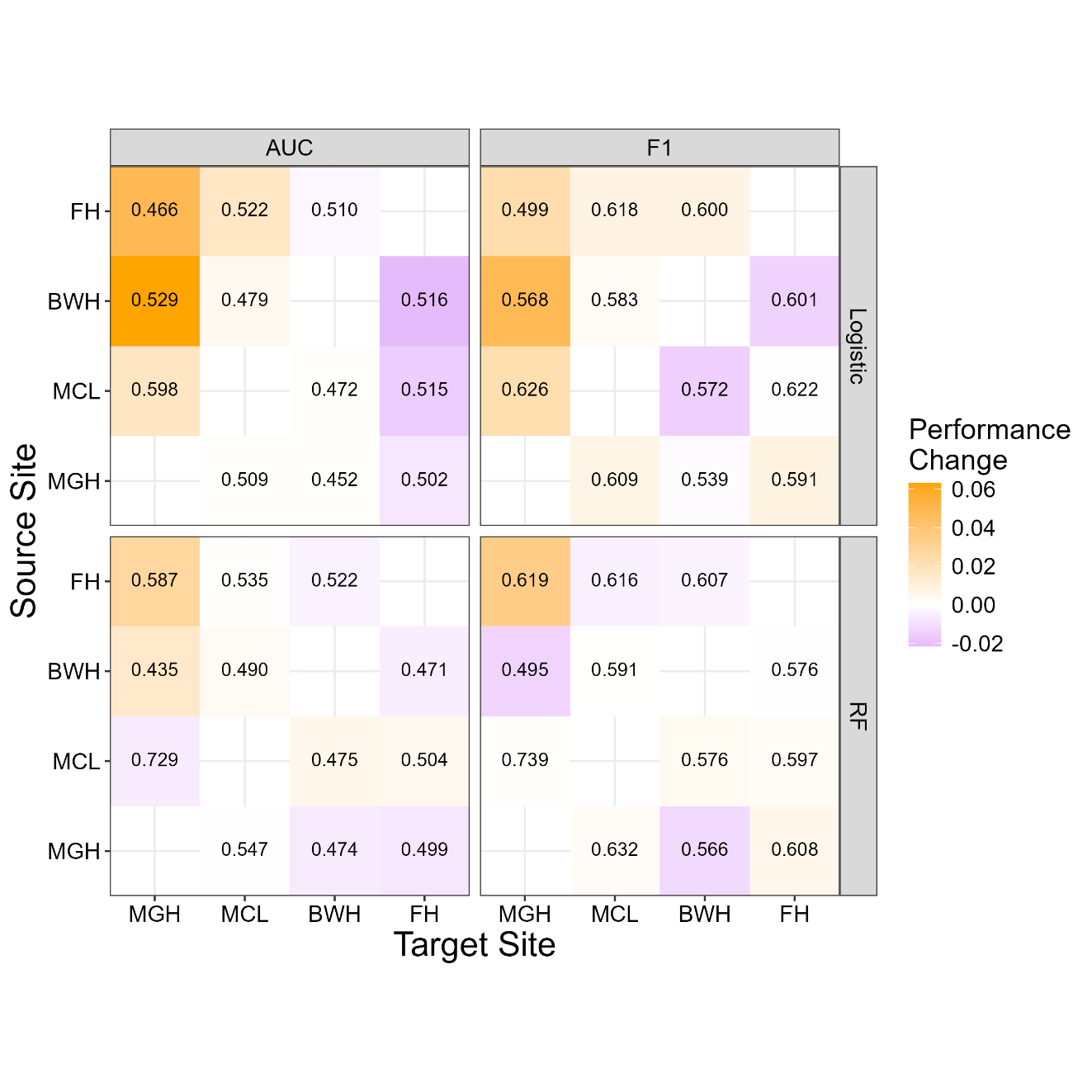


Figure 2: Cross-site prediction performance of the logistic regression models (top) and RF (bottom) with four additional SES variables included after adjusting for covariate shift. The text values represent model performance with covariate shift adjustment, while the tile colors indicate the magnitude and direction of the adjustment’s effect: positive changes in the prediction metric are shown in orange, and negative changes in blue. BWH=Brigham and Women’s Hospital, MCL=McLean, MGH=Mass General Hospital, FH = Faulkner Hospital.
