# Supplementary material for: Cross-Site Predictions of Readmission After Psychiatric Hospitalization With Mood or Psychotic Disorders: Retrospective Study
Source: JMIR Ment Health. 2025 Sep 12;12:e71630. doi: 10.2196/71630 (PMC12431164; doi:10.2196/71630)
Supplement: Multimedia Appendix 2 [file mental-v12-e71630-s002.docx]

#### Formal description of inverse probability weighting algorithm

We first introduce some notation for ease of exposition. Let $(Y,X,S)$ be the triplet indicating the outcome, set of predictors and site index. Denote the loss function of a particular model by $l(Y; f(X))$, where $f$ is the prediction function, and the distribution of $(Y,X)$ in site $S=s$ by $p_{s}$. Under the assumption of covariate shift, we have $p_{s}(y|x)=p_{s'}(y|x)$ but $p_{s}(x)\neq p_{s'}(x)$. The in-site prediction model for site *s* is trained to optimize the expected loss $L(s)=E_{p_{s}}(l(Y; f(X))$. In practice, since $p_{s}$ is unknown, the empirical loss $\hat{L}(s) = \sum_{i=1}^{n_{s}} l(Y_{i}; f(X_{i}))/n_{s}$, which is a consistent estimator of $L(s)$, is used for training. Here $n_{s}$ is the sample size of the training set for site $s$. If we want to build prediction models for site $s'$ based on data of site $s$, we can leverage the following result:

$$E_{p_{s'}}(l(Y; f(X))= E_{p_{s}}[l(Y; f(X))p_{s'}(Y,X)/p_{s}(Y,X)]=E_{p_{s}}[l(Y; f(X))p_{s'}(X)/p_{s}(X)].$$

The last equality follows due to the covariate shift assumption. In other words, this means that we can use the following weighted empirical loss function, based on data in site $s$, to train a prediction function that asymptotically minimizes the expected loss for site $s'$:

$$\hat{L^{tr}}(s')={n_{s}}^{-1}\sum_{i=1}^{n_{s}} l(Y_{i}; f(X_{i}))p_{s'}(X_{i})/p_{s}(X_{i}).$$

Note that the ratio $p_{s'}(X_{i})/p_{s}(X)$ can be estimated using Bayes’ rule:

$p_{s^{'}}(X_{i})/p_{s}(X)=Pr(S=s)/Pr(S=s')\times Pr(S=s'|X)/Pr(S=s|X).$

We estimate the first ratio to the right-hand side by $n_{s}/n_{s'}$ and the second ratio through logistic regression with the binary outcome $I(S=s)$ and covariates $X$ on the merged data of site $s$ and $s'$.
